# The hypothalamus as the central regulator of energy balance and its impact on current and future obesity treatments

**DOI:** 10.20945/2359-4292-2024-0082

**Published:** 2024-11-06

**Authors:** Bruna Bombassaro, Eliana P. Araujo, Licio A. Velloso

**Affiliations:** 1 Universidade de Campinas Centro de Pesquisa em Obesidade e Comorbidades Campinas SP Brasil Centro de Pesquisa em Obesidade e Comorbidades, Universidade de Campinas, Campinas, SP, Brasil

**Keywords:** Food intake, adipose tissue, brain, neuron, proopiomelanocortin, POMC

## Abstract

The hypothalamus is a master regulator of energy balance in the body. First-order hypothalamic neurons localized in the arcuate nucleus sense systemic signals that indicate the energy stores in the body. Through distinct projections, arcuate nucleus neurons communicate with second-order neurons, which are mostly localized in the paraventricular nucleus and in the lateral hypothalamus. The signals then proceed to third- and fourth-order neurons that activate complex responses aimed at maintaining whole-body energy homeostasis. During the last 30 years, since the identification of leptin in 1994, there has been a great advance in the unveiling of the hypothalamic and extra-hypothalamic neuronal networks that control energy balance. This has contributed to the characterization of the mechanisms by which glucagon-like peptide-1 receptor agonists promote body mass reduction and has opened new windows of opportunity for the development of drugs to treat obesity. This review presents an overview of the mechanisms involved in the hypothalamic regulation of energy balance and discusses how advancements in this field are contributing to the development of new pharmacological strategies to treat obesity.

## INTRODUCTION

Obesity affects over 600 million people worldwide. According to projections, these numbers will soon double (www.worldobesity.org), increasing the prevalence of several comorbidities that negatively impact life quality and longevity. Most cases of obesity result from a combination of environmental factors acting upon a permissive genetic landscape. Among these environmental factors, the consumption of calorie-dense foods and a sedentary lifestyle are undisputedly the most important ones and have been widely explored in clinical and experimental studies. Conversely, the genetic component of polygenic obesity is extremely complex and has not been completely elucidated yet. The greatest advancement in this field has been achieved by genome-wide association studies (GWAS), which have identified dozens of genes playing distinct roles in energy balance and potentially predisposing to distinct forms of obesity (1). Interestingly, many of these genes are expressed solely or predominantly in the hypothalamus; moreover, in rare monogenic forms of obesity, virtually all genes identified to date encode proteins with important functions in this region of the brain (2).

**Figure 1:**
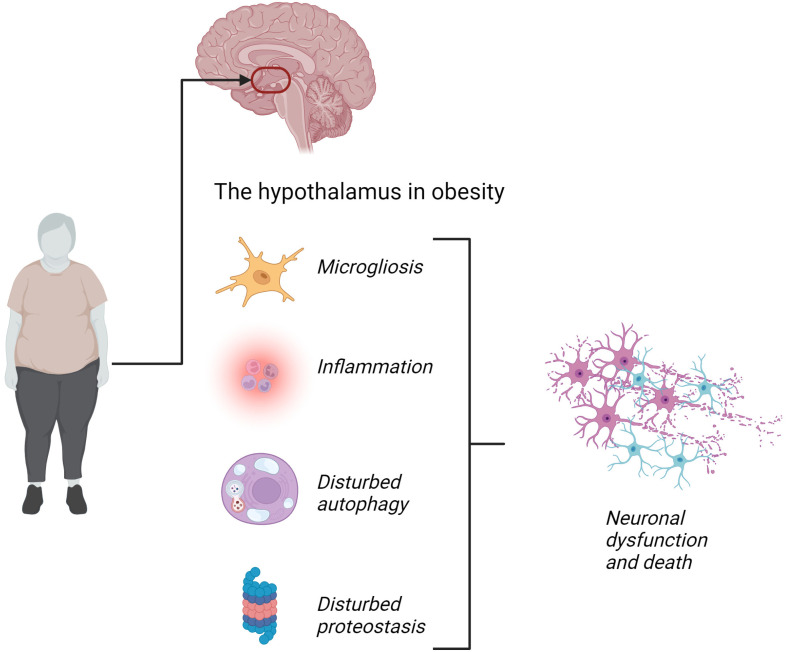
Schematic illustration of the main abnormalities in the hypothalamus in obesity. According to experimental studies, it has been shown that the consumption of excessive dietary fats and, to a lesser extent, excessive dietary sugars can promote hypothalamic microgliosis, inflammation, abnormal regulation of autophagy, and proteostasis, leading to defective function and, eventually, apoptosis of hypothalamic neurons involved in whole-body energy balance. In humans, evidence is based on neuroimaging studies, therefore, it is currently uncertain whether all these abnormalities exist. Thus, this schematic figure is illustrative.

In studies evaluating the impact of dietary factors that promote body mass gain, such as fats and sugars, the hypothalamus has been identified as an important anatomical site exhibiting abnormal function (3). Thus, in experimental models, the consumption of fat-rich diets induces an inflammatory response in the hypothalamus, which rapidly affects the neuronal response to anorexigenic signals, such as leptin and insulin (4,5). This is also true when the dietary intervention is rich in both fats and sugars and even, although in a later stage, only sugars (6,7). The nature of the hypothalamic defects promoted by the excessive consumption of dietary fats is wide and complex, including abnormalities in proteostasis, autophagy, mitochondrial turnover, and RNA granules, which can eventually lead to neuronal apoptosis (8,9). In addition, human studies using different methods of neuroimaging have also shown functional and structural abnormalities in the hypothalamus of individuals with obesity (10,11), which is even more pronounced in those with both obesity and diabetes (12) and can appear very early in life, as shown in a study with pediatric patients (13) (Figure 1).

Recent advancements in the development of drugs to treat obesity have provided further support for the important role played by the hypothalamus in the genesis of the disease and as a therapeutic target. Studies have shown that the glucagon-like peptide-1 (GLP1) receptor (GLP1R) agonists liraglutide and semaglutide act predominantly through the activation of GLP1R in proopiomelanocortin (POMC) neurons of the hypothalamic arcuate nucleus (ARC) to promote their anorexigenic effects (14,15), which can lead, in the case of semaglutide, to body mass reductions of up to 15% over 6 months of treatment (16). Thus, combining the results obtained from genetic, dietary, and pharmacological studies, the hypothalamus has emerged as the most important target for the development of drugs to treat obesity. This short review explores the hallmarks of the research that provided evidence for supporting the important role of the hypothalamus in obesity.

## LEPTIN AND THE HYPOTHALAMIC NEUROCIRCUITS THAT REGULATE ENERGY BALANCE IN THE BODY

The identification of leptin in 1994 is a hallmark of the research in the field of whole-body energy balance (17), as the characterization of its sites and mechanisms of action has played a central role in unveiling the complex neuronal networks that regulate caloric intake and energy expenditure (18). Hypothalamic neurons located in the ARC are the primary targets of leptin action (18). There are two main subpopulations of neurons in this region; the POMC and the agouti-related peptide (AgRP) neurons, which have antagonistic actions in response to the systemic signals that indicate energy storage in the body (19). During periods of fasting or body energy depletion, the reduction in leptin signaling, associated with increased ghrelin action, activates AgRP neurons, which in turn act through three overlapping mechanisms to increase appetite: (A) antagonism of melanocortin 3 receptor (MC3R) and melanocortin 4 receptor (MC4R) in the paraventricular nucleus of the hypothalamus (PVH), (B) inhibition of PVH anorexigenic neurons by neuropeptide Y (NPY), and (C) inhibition of PVH anorexigenic neurons by gamma-aminobutyric acid (GABA) (19). The importance of AgRP in energy balance has been demonstrated in transgenic mice that either do not express this peptide or express it in increased amounts. Mice lacking AgRP presented an important reduction in feeding (20), whereas those with increased expression of AgRP developed hyperphagia and obesity (21). Leptin and ghrelin provide the most robust signals controlling AgRP-neuron function (22,23). In the presence of leptin, which indicates body energy surplus, AgRP neurons are inhibited, thus facilitating the activation of melanocortin receptors in second-order neurons (22). Conversely, during periods of fasting, there is increased production of ghrelin in the stomach, which results in hypothalamic signals that activate AgRP neurons, thus promoting hunger (23).

Initial studies characterizing leptin actions in the ARC suggested the existence of a homogeneous subpopulation of POMC neurons involved primarily in the reduction of food intake (24). However, recent studies employing single-cell RNA sequencing strategies provided a major shift in the understanding of ARC POMC identities and physiology (25,26). Currently, at least seven distinct POMC subpopulations are believed to exist in the ARC (25). Some are not responsive to leptin, and others are not primarily involved in regulating food intake (27). According to the expression of certain surface receptors, these distinct POMC subpopulations can respond to leptin, insulin, GLP1, or serotonin, and in response to these signals, they play a role in the independent regulation of food intake, systemic glucose handling, locomotion, and behavior (27). Nevertheless, the importance of the melanocortin system, in particular of POMC neurons, in energy balance is evidenced by the impact on body mass promoted by mutations of genes that encode proteins of this system (1,2). Thus, mutations of the gene encoding for MC4R represent the most prevalent form of monogenic obesity, reaching up to 4% of all cases of severe obesity (1,2). In addition, mutations in the *POMC* gene and in the *PCSK1* gene, which encodes an endopeptidase that plays a critical role in POMC processing, also result in severe forms of obesity (1,2).

The PVH is one of the most important sites receiving projections from ARC AgRP and POMC neurons (28). Some PVH neurons express MC4R, which responds to direct stimulation of ARC neurons (28). This system has been shown to be both sufficient and necessary for regulating food intake in response to the activation of POMC and AgRP ARC neurons (29). However, contrary to initial belief, the melanocortin system in the PVH is not involved in regulating energy expenditure. This effect is mediated by cholinergic preganglionic sympathetic neurons in the intermediolateral nucleus of the spinal cord, which also express MC4R and respond to feeding/fasting cycles and to cold exposure (30). The lateral hypothalamus (LH) also receives projections from ARC AgRP and POMC neurons. The main outcomes of the melanocortin system activity in neurons of the LH are related to the control of hedonic feeding by connections with the ventral tegmental area (31); sleep/arousal cycles, mediated by connection with the locus coeruleus (32); and locomotor activity, mediated by a combination of connections with the nucleus accumbens and the ventral tegmental area (33).

The lateral parabrachial nucleus (LPBN) has emerged as yet another brain region receiving projections from the hypothalamus that are important for the regulation of feeding (34). In this case, the projections originate in the PVH and play an important role by acting as a hub that connects metabolic signals from the hypothalamus with the signals originating in the gastrointestinal tract, thus reflecting the transit, digestion, and absorption of nutrients in the gut (34,35).

## OTHER PLAYERS IN THE HYPOTHALAMIC REGULATION OF ENERGY BALANCE

The energy status of living cells must be under strict control, promoting a balance between catabolic and anabolic pathways. Some metabolic enzymes are capable of directly sensing changes in the AMP:ATP ratio, such as enzymes responsible for glycogen breakdown or glycolysis, but the main energy sensor within the cells is AMP-activated protein kinase (AMPK). Once activated, AMPK positively regulates the cellular process involved in catabolic pathways and inhibits anabolic metabolism to guarantee the production of ATP (36). As for its role in energy balance, AMPK regulates energy pathways in tissues that are important in controlling energy, such as the liver, pancreas, and hypothalamus. Its expression on hypothalamic nuclei is particular for each subunit (α, β, and γ) but, as a whole, it is present in all hypothalamic regions (37). The detailed evaluation of the function of AMPK in distinct hypothalamic regions and neuronal subpopulations has been explored in cell-specific transgenic mice. The deletion of AMPK in POMC neurons reduces energy expenditure and increases fat mass in mice, whereas its deletion in AgRP neurons promotes anorexigenic effects (38). In 2012, AMPK was described to be involved in estradiol effects on the ventromedial nucleus of the hypothalamus (VMH), increasing thermogenesis in the brown adipose tissue. Again, in the VMH, AMPK inhibition protected against obesity by increasing thermogenesis and improving lipid and glucose metabolism, in this case, acting in steroidogenic factor 1 (SF1) neurons. Additionally, AMPK has been implicated in a carbohydrate meal preference in refeeding due to its activation during fasting in corticotropin-releasing hormone (CRH) PVH neurons (39).

Peripheral cues are essential for the control of energy status and metabolism by the central nervous system. Hormones such as insulin, leptin, and ghrelin are well established in exerting central signals in the hypothalamus to control food intake; however, they are only part of these cues, which include signals such as visual and odor, taste, and nutrient sensing (40). During fasting, the olfactory capacity is heightened compared with satiety, and the simple detection of food smell shifts hypothalamic neuronal circuits, inhibiting AgRP and activating POMC neurons within seconds. The ablation of olfactory sensory neurons in lean mice elicits resistance to obesity and increased sympathetic tonus leading to thermogenesis, but if this loss occurs after obesity development, the reduced olfactory activity increases fat mass and insulin resistance. For example, the genetic ablation of olfactomedin 2 (OLFM2), a secretory glycoprotein, reduces food intake, increases brown adipose tissue thermogenesis, and improves insulin resistance; conversely, its overexpression in the lateral hypothalamic area (LHA) has opposite effects (41).

## HYPOTHALAMIC FUNCTIONS OF GENES INVOLVED IN MONOGENIC AND POLYGENIC OBESITY

The *MC4R* gene is undisputedly the most important gene involved in monogenic obesity, and *MC4R* variants are commonly found in studies exploring polymorphisms in non-monogenic forms of obesity (1,2). This gene encodes a G protein-coupled receptor that is expressed not only in the hypothalamus but also in several other brain regions. The alpha-melanocyte-stimulating hormone (*a*-MSH) is a natural ligand for MC4R, and its binding to the receptor induces intracellular signaling through cyclic AMP. In the hypothalamus, the activation of MC4R results in a potent anorexigenic response. Humans and experimental models with impaired MC4R function develop severe obesity and present an increased predisposition to comorbidities (42).

Mutations or polymorphisms of the leptin and leptin receptor genes have been identified in both monogenic and polygenic forms of obesity (43). The activation of the leptin receptor in ARC neurons promotes signal transduction through signal transducer and activator of transcription 5 (STAT5) and protein kinase B (Akt) systems. In POMC neurons, this signal results in increased expression of POMC and enzymes needed to process POMC into *a*-MSH, whereas in AgRP neurons, it has an inhibitory action. Thus, the net result of the activation of the leptin system in the hypothalamus is the activation of the melanocortin system leading to an anorexigenic response. Both humans and experimental models with mutations of these genes present extreme hyperphagic obesity with disturbed reproduction and predisposition to diabetes and hypertension (43).

Mutations or polymorphisms of *PCSK1* have been reported both in monogenic and polygenic forms of obesity. This gene encodes a member of the subtilisin-like proprotein convertase family – prohormone convertase 1/3 – which is involved in the processing of several proneuropeptides and prohormones (44). In hypothalamic POMC neurons, it catalyzes distinct steps of the POMC processing that lead to the production of *a*-MSH and *b*-endorphin. Humans with loss-of-function *PCSK1* mutations may present a syndrome that comprises obesity, hypogonadotropic hypogonadism, diarrhea, abnormal thyroid and adrenal function, and impaired regulation of systemic glucose levels (44).

Mutations in the *SIM1* gene have been reported in some patients with severe monogenic obesity, but in polygenic obesity, *SIM1* polymorphisms are rare (45). Notably, *SIM1* encodes a basic helix-loop-helix transcription factor that plays a critical role in the development of the PVH (46). Mice homozygous for a null allele of *SIM1* lack PVH and die perinatally. However, *SIM1* heterozygous mice are viable and develop severe hyperphagic obesity, with hyperleptinemia and hyperinsulinemia (46).

Notably, GWAS have identified polymorphisms of the fat mass and obesity-associated (*FTO*) gene as the most prevalent risk alleles for obesity (47). Indeed, *FTO* is highly expressed in the hypothalamus, and according to a study, FTO intronic long-range enhancers regulate iroquois-class homeobox protein 3 (*IRX3*) expression (48). Notably, *IRX3* is expressed in hypothalamic POMC neurons and is regulated by dietary interventions. Alterations of its expression can promote changes in adiposity by modifying food intake and energy expenditure (49).

Taken together, the results from studies of both monogenic and polygenic obesity place the hypothalamus in an important position, both as an anatomical site responsible for the development of the disease and as a target for current and future therapeutic interventions.

## HYPOTHALAMIC CELLULAR ABNORMALITIES IN OBESITY

The consumption of a high-fat diet, as well as a high-fat/high-sugar diet, triggers an inflammatory response in the hypothalamus (3). Similar to inflammation that occurs in other regions of the body, in the hypothalamus, diet-induced inflammation has acute and chronic phases with distinct functional and structural outcomes (50). In experimental models of obesity, exposure to harmful dietary factors for up to 15 days leads to an acute inflammatory response, whereas longer periods of dietary exposure lead to a chronic inflammatory phase (50).

The hypothalamus has been shown to respond with increased expression of chemokines and inflammatory cytokines as early as 1 day after the introduction of a high-fat diet (11,51). These substances are mostly produced by microglia and, upon action in neighboring neurons, can activate intracellular signal transduction through the c-Jun N-terminal kinase (JNK) and nuclear factor-κB (NFkB) pathways (4). At 3 days after the beginning of exposure to the diet, the activation of these inflammatory pathways leads to the installation of neuronal resistance to the actions of the energy homeostatic hormones leptin and insulin. In the first week of exposure to harmful dietary factors, microglia and astrocytes undergo structural and functional changes. This includes astrocyte and microglia increase in gene expression of glial fibrillary acidic protein (GFAP) and EGF-like module-containing mucin-like hormone receptor-like 1 (Emr1), which is followed by increased microglia and astrocyte cell numbers (11). A recent study investigated the details of the initial hours of exposure to a high-fat diet and demonstrated that GFAP transcripts increased 1 hour after the introduction of the diet, which may be the earliest dysfunctional outcome resulting from the consumption of large amounts of dietary fats, whereas ionized calcium-binding adaptor molecule 1 (Iba1) increased after 3 hours. Histological analysis revealed that astrocytes and microglia initially undergo morphological changes, whereas cell proliferation occurs at a later point (52). Importantly, there is also recruitment of T lymphocytes to the hypothalamus, suggesting the emergence of a response with specificity and robustness (53).

Other important features of the acute phase of diet-induced hypothalamic inflammation are the activation of the unfolded protein response and the disruption of physiological mitochondrial turnover, providing additional stress to the hypothalamic neurons, and directly impacting their capacity to respond properly to hormones and nutrients (5,54). Reduction of hypothalamic mitochondrial respiratory function is present several weeks after the beginning of dietary intervention; however, there is an alteration in mitochondrial molecular structure as early as 1 day after exposure to a high-fat diet (55). This is accompanied by changes in the expression of the chaperones GRP78 and GRP94 that increase rapidly after dietary intervention, suggesting that fatty acid overload negatively impacts the physiology of the endoplasmic reticulum (55). Despite the important role played by the impaired functions of mitochondria and endoplasmic reticulum in the early phase of diet-induced hypothalamic abnormalities, most of the effects on these organelles are secondary to the early inflammatory response triggered by the dietary fats (55).

Most of the hypothalamic abnormalities developed during a short period of consumption of a high-fat diet are reversible, as far as the experimental animals are returned to regular chow before 8 weeks of dietary intervention (56). However, upon prolonged exposure to a high-fat diet, chronic inflammation will result in profound and apparently irreversible damage. As in other chronic inflammatory conditions, chronicity in the hypothalamus leads to vascular proliferation and damage, which, in the particular case of the hypothalamus, results in injury to a very unique type of blood-brain barrier (BBB). In the medium eminence-ARC interface, there is a special type of BBB that, differently from most regions of the brain, presents a leakier function that is physiologically important as the neurons sitting in the ARC are programmed to sense small oscillations in the blood levels of nutrients and hormones, and this is facilitated by a leakier BBB. However, the diet-induced inflammation of the hypothalamus leads to an even increased leakiness of the BBB, allowing the entrance of larger amounts of circulating fatty acids that boost the ongoing inflammatory activity and, thus, increase hypothalamic damage (50,57).

Hypothalamic microglia also undergo chronic changes during prolonged consumption of a high-fat diet. During the initial phase of exposure to dietary fats, resident hypothalamic microglia undergo structural and functional changes; however, the persistence of exposure to the harmful components of the diet activates chemotaxis and bone marrow-derived monocytes migrate to the mediobasal hypothalamus (MBH) (58). Surprisingly, a recent study from our group has shown that, counterintuitively, the migrating monocytes express predominantly anti-inflammatory factors and could act in the hypothalamus to mitigate the damage produced by the prolonged consumption of dietary fats. In addition, the study identified a considerable degree of sexual dimorphism in the transcriptional signatures of migrating monocytes (59).

Experimental studies have identified several abnormalities in ARC neurons of mice chronically fed a high-fat diet, including defective regulations of autophagy, mitophagy, proteostasis, RNA granules, and even increased apoptosis (8). As a whole, the neuronal abnormalities seen in the hypothalamus of chronically obese mice have similarities with the neuronal changes present in models of neurodegenerative diseases, such as Parkinson's and Alzheimer's (8). This may explain, at least in part, the high degree of therapeutic refractoriness and recurrence of obesity.

## EVIDENCE OF HYPOTHALAMIC ABNORMALITIES IN HUMAN OBESITY

Because of anatomical constraints, human studies evaluating the hypothalamus in obesity have been mostly performed using neuroimaging methods. Using conventional magnetic resonance, studies measuring the T2 relaxation time of the MBH have revealed that gliosis is present in a magnitude that has a direct correlation with body mass index (11). This finding is more severe in patients with obesity and diabetes than in those with obesity without diabetes (12). Moreover, this is a hypothalamic-specific structural abnormality, since evaluations performed in other brain regions were not capable of detecting similar defects. Importantly, hypothalamic gliosis appears early during the development of obesity, as it is also present in the hypothalamus of pediatric patients with increased adiposity (13). Using another approach (functional magnetic resonance), studies have demonstrated a reduction of glucose- and cold-induced signals in the hypothalamus of patients with obesity (60,61). Moreover, in patients with obesity submitted to bariatric surgery, body mass reduction was accompanied by a partial recovery of the abnormal hypothalamic response to glucose, suggesting that, at least in part, this is a reversible defect (10). One study that evaluated hypothalamic specimens obtained postmortem showed that gliosis increased and correlated with T2 relaxation time in the MBH, therefore validating the use of magnetic resonance imaging for the detection of gliosis in the hypothalamus of humans (62). A recent article has provided a broad review of the studies evaluating the hypothalamic function and structure in humans with obesity (63).

## HYPOTHALAMIC MECHANISMS OF ACTION OF GLP1 RECEPTOR AGONISTS

Early studies exploring the biological actions of GLP1 have identified the presence of its receptor in the hypothalamus, suggesting that GLP1 could be involved in regulating metabolic and/or autonomic functions (64). However, it was only after the identification of exendin-4 that the anorexigenic and body mass reduction effects of ligands of the GLP1R were unveiled (65). Early studies performed in rodents were promising, but one of the first clinical trials reported a body mass loss of only 2 kg after up to 2 years of exenatide intervention (66), leading to some frustration. Shortly after the disappointing results obtained with exenatide, a study showed excellent results with liraglutide, with body mass reduction of 9 kg in 6 months (67), opening a new and promising window of opportunities for the treatment of obesity.

The main effect of liraglutide is the reduction of caloric intake, and this was suspected to be due to its actions in the brain. To explore this hypothesis, mice were treated with fluorescently labeled liraglutide and its binding sites were evaluated using a combination of tissue clarification and three-dimensional fluorescent microscopy (14). The beautiful images obtained with this method provided the first and most convincing evidence for the site of action of liraglutide in the ARC (14). Further experiments using transgenic mice expressing fluorescent protein in specific subpopulations of hypothalamic neurons revealed that liraglutide acted primarily by binding to GLP1R in POMC neurons, with no interaction with NPY/AgRP neurons. Moreover, it was shown that a third of the GABAergic neuronal population was involved in the mechanism of action, by providing an inhibitory loop to the NPY/AgRP neurons (14).

The development of semaglutide provided yet another breakthrough in the treatment of obesity, with clinical trials showing up to 14 kg of body mass reduction after 1 year (16). In a similar way to liraglutide, the main effect of semaglutide was the reduction of caloric intake, and this raised the question about the mechanisms that could explain the superiority of semaglutide. Using experimental approaches similar to the ones used originally to evaluate the mechanisms of action of liraglutide, it was shown that semaglutide was capable of reaching ARC neurons with greater efficiency than liraglutide (15). It was also shown that projections from the primary sites of action of semaglutide reached brain regions involved in meal termination, such as the amygdala and the solitary tract nucleus (15).

Liraglutide and semaglutide were not primarily developed for the treatment of obesity, nor was the hypothalamus regarded as their primary target. Nonetheless, both experimental and clinical studies provided the proof-of-concept that placed these drugs as pioneers in the new era of the pharmacological treatment of obesity. Moreover, their actions in the central nervous system consolidated the hypothalamus as an important target to treat obesity, reinforcing the evidence provided in genetic and mechanistic studies.

## THE FUTURE OF OBESITY PHARMACOLOGICAL THERAPY – IS THE HYPOTHALAMUS THE BEST TARGET?

Ideally, the treatment of obesity should be based on a combination of reduced caloric intake and increased energy expenditure. Notably, GLP1R agonists act by reducing caloric intake only. In the case of semaglutide, there is evidence suggesting that it also mitigates the reduction of energy expenditure that follows body mass reduction (15). No currently approved medication acts primarily by increasing energy expenditure. Thus, future development in this field should seek pharmacological approaches that either can reduce caloric intake and increase energy expenditure with one single drug or combine drugs to achieve such results.

Hypothalamic neurons play very important roles in controlling both caloric intake and energy expenditure. However, contrary to initial expectations, the hypothalamic neuronal populations that control each of these functions differ, suggesting that finding a single drug capable of controlling both responses simultaneously may be a difficult task. Thus – as in other multifactorial complex diseases, such as diabetes, hypertension, and atherosclerosis – the combination of drugs with distinct mechanisms of action may lead to the best results in obesity.

An alternative approach that has provided recent pharmacological and therapeutic advances was the development of single molecules capable of activating distinct receptors. This new class of drugs, currently known as dual or triple agonists, are designed to interact with two or three receptors, therefore boosting their pharmacological effects (68). Tirzepatide was the first drug of this class approved for human use, with an initial indication for the treatment of type 2 diabetes. However, phase III clinical trials have demonstrated its potent action in reducing body mass, and its use in patients with obesity was recently approved by the FDA (69). Tirzepatide is a dual GLP1 and gastric inhibitory polypeptide (GIP) agonist. So far, the mechanisms of action attributed to tirzepatide are the increase of insulin production and its release by the pancreatic islets and the increased production of the insulin-sensitizing hormone adiponectin (70). To date, no study has evaluated the actions of tirzepatide in the hypothalamus; however, considering the magnitude of body mass reduction obtained with its use, the activation of POMC neurons is an expected outcome.

In addition to targeting GLP1 and GIP receptors, other drugs in development target the glucagon receptor and amylin receptor. Based on experimental data, it is currently known that all these four targets are expressed in the hypothalamus and their activation leads to reduced food intake. Thus, these data provide additional support for the importance of the hypothalamus as a therapeutic target in obesity.

Unfortunately, little advance has been achieved in the field of increased energy expenditure. No drug in an advanced stage of development acts through the hypothalamus to increase energy expenditure. A promising venue is an indication that β3-adrenergic receptor activation in brown adipose tissue results in increased energy expenditure, which could contribute to body mass reduction. However, drugs available at this moment have severe cardiovascular side effects that impede their clinical use.

In conclusion, the hypothalamus is an excellent target for drugs that induce reduced caloric intake. Hypothetically, it could also be a good target for drugs that stimulate energy expenditure. However, at this moment, it seems that stimulating energy expenditure could be achieved more easily by drugs acting in peripheral organs, such as brown adipose tissue. Nevertheless, recent advances in the therapeutics of obesity have placed the hypothalamus in a very important position as a drug target, and this position seems to be definitive.
